# Bibliography

**Published:** 1886-06

**Authors:** 


### BIBLIOGRAPHY.

A REFERENCE HANDBOOK OF THE MEDICAL SCIENCES, embracing the entire range of *Scientific and Practical Medicine* and *Allied Sciences*, by various writers; illustrated by *chromo-lithographs* and wood engravings; edited by Albert H. Buck, M. D., Vols. 1 & 2. William Wood & Company, New York, 1886.

We have received Vols. 1 and 2 of this masterpiece of painstaking, laborious and successful compilation. It is, strictly speaking, a complete encyclopedia of medicine and surgery; brought up to the moment of going to press; and contains exhaustive and well written treatises on every subject connected with the science and arts of medicine and surgery, by the masters of modern art. It is practically *invaluable*. Possessed of this complete set, the practitioner should not want any other library. The subjects are arranged alphabetically; the first two volumnes giving from "ace-cat" to "eye," and embracing every subject under those heads. The article on cataract alone, is worth the price of the set; while that on Electricity in Surgery, puts the reader in possession, practically, of all the facts at present known with reference to this important therapeutic advance. Indeed, the work is a masterpiece, and no live man can afford to do without it. The chromo-lithographs are models of the art, while the abundant wood cuts are above the average. Bound in a neat and substantial morocco, sold by subscription only; for the complete set, seven volumns, $8, per volume.

*Text Book of Ophthalmoscopy*, by Edward G. Loring, M. D., part 1: The Normal Eye; Determination of Refraction;

Disease of the Media; Physiological Optics and Theory of the Ophthalmoscope; N. Y., D. Appleton & Co., 1, 3 & 5 Bond Street, 1886.

To say that this contribution to the subject of Ophthalmoscopy is a valuable addition would be but scant justice; it is a big advance on anything heretofore offered to the profession, that has fallen under our observation. The general practitioner shrinks from diving into the mystery of thorough ophthalmoscopy, as something unusually difficult; but with the introduction of the ophthalmoscope, much that was mysterious and little understood, has been elucidated, and is now relegated to the domain of demonstrated fact. Dr. L. has rendered no little service to his brethren by beginning at the beginning, and teaching them *how to use* the ophthalmoscope, so as to get the benefit of its revelations. Both the direct and indirect methods of using the instrument are fully and comprehensively described; and the value of such knowledge will be apparent, when we say,—quoting the author, "Five-sixths of the art of ophthalmoscopy are contained in a knowledge of the normal eye; the rest is a series of representations which can be read almost at sight." Want of room prevents our saying more of this truly meritorious work. The lithographic plates are superior pieces of work, and the book is turned out with that neatness which characterizes the Appletons. To the specialists, particularly, this work must be of great assistance, while even to the medical tyro, it could not prove otherwise than interesting.

